# Sperm Limitation Produces Male Biased Offspring Sex Ratios in the Wasp, *Nasonia vitripennis* (Hymenoptera: Pteromalidae)

**DOI:** 10.1093/jisesa/ieac032

**Published:** 2022-06-28

**Authors:** Z G Holditch, K N Ochoa, S Greene, S Allred, J Baranowski, S M Shuster

**Affiliations:** Department of Biological Sciences, Northern Arizona University, Flagstaff, AZ 86011, USA; Department of Biological Sciences, Northern Arizona University, Flagstaff, AZ 86011, USA; Department of Biological Sciences, Northern Arizona University, Flagstaff, AZ 86011, USA; Department of Biological Sciences, Northern Arizona University, Flagstaff, AZ 86011, USA; Department of Biological Sciences, Northern Arizona University, Flagstaff, AZ 86011, USA; Department of Biological Sciences, Northern Arizona University, Flagstaff, AZ 86011, USA

**Keywords:** local mate competition, sex allocation, Hymenoptera, parasitoids, behavior

## Abstract

Haplo-diploid sex determination in the parasitoid wasp, *Nasonia vitripennis* (Walker), allows females to adjust their brood sex ratios. Females influence whether ova are fertilized, producing diploid females, or remain unfertilized, producing haploid males. Females appear to adjust their brood sex ratios to minimize ‘local mate competition,’ i.e., competition among sons for mates. Because mating occurs between siblings, females may optimize mating opportunities for their offspring by producing only enough sons to inseminate daughters when ovipositing alone, and producing more sons when superparasitism is likely. Although widely accepted, this hypothesis makes no assumptions about gamete limitation in either sex. Because sperm are used to produce daughters, repeated oviposition could reduce sperm supplies, causing females to produce more sons. In contrast, if egg-limited females produce smaller broods, they might use fewer sperm, making sperm limitation less likely. To investigate whether repeated oviposition and female fertility influence gamete limitation within females, we created two treatments of six mated female wasps, which each received a series of six hosts at intervals of 24 or 48 h. All females produced at least one mixed-sex brood (63 total broods; 3,696 offspring). As expected, if females became sperm-limited, in both treatments, brood sex ratios became increasingly male-biased with increasing host number. Interhost interval did not affect brood size, total offspring number, or sex ratio, indicating females did not become egg limited. Our results support earlier studies showing sperm depletion affects sex allocation in *N. vitripennis*¸ and could limit adaptive sex ratio manipulation in these parasitoid wasps.

Parasitoid wasps provide a model invertebrate system for investigating offspring sex-ratio manipulation by females, particularly for studies of Hamilton’s Local Mate Competition (LMC) hypothesis (King 1992). According to this hypothesis, populations in which mating readily occurs between siblings will favor sex ratios (i.e., proportion males) that maximize production of grand-offspring by reducing mate competition between sons (Hamilton 1967, p. 481). Werren (1980) suggested female *Nasonia vitripennis* Walker (Hymenoptera: Pteromalidae) optimize mating opportunities in a manner consistent with LMC by adjusting their brood sex ratio in response to the presence of other ovipositing females within a host patch. To reduce mate competition among her sons, Werren (1980, 1983, 1984) predicted that the first female to oviposit on a host (primary female) would produce a strong daughter bias. In contrast, the second female to oviposit on a host (secondary female) was expected to increase the proportion of sons produced to match the intensity of LMC her sons would experience given the clutch size of the primary female and the number of other ovipositing females. An extensive literature describes the adaptive outcomes of LMC in parasitoids (reviewed in Pannebakker et al. 2013). 

The LMC model assumes that female parasitoids influence the production of offspring of either sex and can do so in a precise manner (Orzack 1986, Orzack and Parker 1986, and Pannebaker et al. 2013). Haplo-diploid sex determination in wasps provides a mechanism for sex ratio adjustment because females may influence whether ova are fertilized (producing diploid females) or not (producing haploid males; Cook 1993). Female control over offspring sex ratios can be influenced by bacterial symbionts (Werren et al. 2008) and selfish genetic elements that enhance their own transmission by distorting the sex of their hosts’ offspring (reviewed in Stouthamer et al. 2002, Werren and Stouthamer 2003, Akbari et al. 2013, Aldrich et al. 2017). However, two other factors, perhaps more likely to disrupt sex ratio manipulation in female wasps include: 1) sperm limitation within mated females, due to repeated oviposition (e.g., references in King 1987, Perez-Lauchaud and Hardy 1999, King 2000, and thus variable sperm quality e.g., Henter 2004, Ruther 2009), and 2) egg limitation due to variation in female size, which could mitigate limited sperm supplies when female brood sizes are small.

Sperm-limited females in *N. vitripennis* are known to produce fewer daughters in primary ovipositions (King and D’Souza 2004, Boulton and Shuker 2015b, Chirault et al. 2019. However, this form of disrupted or ‘constrained’ sex allocation is thought to be rare in natural populations where mating occurs locally, because strongly male-biased sex ratios would seldom maximize the number of daughters produced by each brood (Hardy and Godfray 1990, Hardy 1994, King and D’Souza 2004, Boivin 2013). Specifically, LMC theory predicts that the fraction of constrained females in wild populations will be small due to balancing selection on population sex ratios (Godfray 1990, Hardy and Godfray 1990). Under these conditions, high frequencies of constrained females are expected to cause a surplus of males within a population, favoring those females who acquire enough sperm to balance the sex ratio by producing more daughters. This prediction is supported by investigations of LMC mating systems in which brood sex ratios typically contain sufficient numbers of males to inseminate all females (Martel et al. 2016). Nevertheless, in wild populations of *N. vitripennis*, as many as 18% of females are depleted of sperm (Skinner 1983). In other parasitoid species, up to 29% of females in natural populations may be sperm-depleted (Hardy and Godfray 1990). These patterns suggest limited sperm supplies exert a nontrivial influence over sex allocation in parasitoids within natural environments.

Egg limitation in *N. vitripennis* may also affect the likelihood of sperm limitation by influencing the rate of sperm use (e.g., Boulton et al. 2015, Kasamatsu and Abe 2015). In parasitoids, egg development occurs in one of two ways: In proovigenic parasitoids, females emerge from their host with their entire egg supply (Flanders 1950, Jervis et al. 2001). In synovigenic parasitoids, such as *N. vitripennis* (Pannebakker et al. 2013) females emerge with some mature eggs, but develop most of their eggs throughout their lives by feeding on hosts. Synovigenic females could experience egg limitation if hosts are scarce, as the lack of nutrition causes females to reabsorb ova (King et al. 1968). However, egg limitation could also arise in host-rich environments, since females oviposit more frequently and thus have less time between hosts to mature eggs (Heimpel et al. 1998, Rosenheim et al. 2000). Egg-limited females could oviposit fewer eggs per host, and so utilize fewer sperm. Under such conditions, females who become egg-limited from frequent oviposition may be less likely to become sperm limited than females who produce larger broods.

Here, we investigated how repeated oviposition and variable brood size may influence brood sex ratio in *N. vitripennis* females. In our experiments, we explored sperm limitation in females by allowing wasps to oviposit repeatedly on *Sarcophaga bullata* pupae. Additionally, we explored egg limitation in females by varying the interval between oviposition bouts (24 vs 48 h). We predicted that when mating period and the effects of variable clutch size are controlled, female wasps whose sperm supplies are limited will produce brood sex ratios that are increasingly male-biased. We also predicted that sperm limitation will be less likely to impact sex allocation in egg-limited females whose average brood size is small. Results consistent with our predictions could indicate that sperm limitation and egg limitation interact to influence “adaptive” sex ratio manipulation in parasitoids.

## Materials and Methods

We used the scarlet eye strain of *N. vitripennis* (Ward’s Science, Rochester NY) in our experiments. Ward’s reports this strain to be derived from and occasionally outcrossed with wasps from outside sources, but once established in-house, their breeders tend to culture within lineage, making the potential for inbreeding high. We note that our use of this strain could limit our conclusions, depending on how the selective environment these wasps experience with respect to mating and sperm storage differs from that found in nature. Given that extreme inbreeding via sib-mating immediately after eclosion is the sine qua non of parasitoid wasp mating systems, we considered this possibility an acceptable risk (see also Discussion).

Nevertheless, to minimize possible negative effects of inbreeding, to minimize maternal effects, and to standardize the relatedness among our experimental wasp stocks, we imposed two rounds of outbreeding before evaluating our results. Specifically, we isolated the Ward’s wasps as 12 d pupae and allowed them to emerge as adults at 25°C for approximately 48 h. Then we paired each parental generation (P) female with one haphazardly selected P generation male from a different host pupa for 18 h for mating. Following the mating period, we separated males from females, and provided each female with a host pupa for 24 h. We then removed these parasitized host pupae and stored them at 25°C for 12 d to create our F_1_ generation. Each day, wasps received ad libitum sugar-water on a cotton swab.

We removed F_1_ wasp pupae from hosts and again created nonsibling male–female pairs for 18 h (same conditions as above). We then isolated males from the presumably-mated females for 24 h. To determine whether repeated oviposition influenced female brood sex ratios, we allowed each mated female to oviposit on six hosts in succession. To determine whether differences in host availability may influence egg limitation in females, we introduced a new host to each mated female every 24 h (*N* = six females) or every 48 h (*N* = six females) for a total of six hosts per female. After 12 d of larval development at 25°C, we counted the number of F_2_ male and female wasp pupae in these hosts. We only included the broods of F_1_ females with at least one host that produced at least one son and one daughter in our analyses. To determine whether repeated oviposition caused females with higher fertility to deplete their supplies of stored sperm more than females with lower fertility, we recorded the total fertility of all females.

We used *S. bullata* pupae used as hosts in this experiment sourced from either Ward’s Science (Rochester NY) or from a colony we established from these pupae (following protocols in Baranowski et al. 2015, Werren and Loehlin 2009). All host pupae were stored at 5°C throughout the experiment. We combined commercial and stock pupae and distributed these hosts haphazardly to females.

### Statistical Analysis

To determine whether repeated oviposition and differences in host availability influenced female brood sex ratio, we used a general linear mixed model (GLMM) to examine whether host number (i.e., the total number of hosts on which females successfully oviposited) and interhost interval (24 or 48 h), with all possible interactions, influenced brood sex ratio. If the full model, containing all fixed-effects was significant, we removed nonsignificant effects from the analysis in a step-wise manner to identify significant influences on brood sex ratio. Sex ratio was fitted to a binomial error distribution and a logit link function. We accounted for individual-level variation by treating female identity as a random effect (following Chirault et al. 2019). To determine whether certain females had contributed disproportionately to our sex ratio estimates, we partitioned the total variance in the sex ratios of families produced by females into within- and among-female components and examined the fraction of the total variance that existed among females. We used a goodness of fit test to compare our sex ratio data to a Poisson distribution.

To determine whether females with higher fertility deplete their supplies of stored sperm more than females with lower fertility, we used a second GLMM to assess the effect of total female fertility (i.e., total number of offspring produced by a female during her lifetime) on the proportion of males produced by females in their final oviposition. As above, sex ratio was fitted to a binomial error distribution and a logit link function.

To determine if repeated oviposition and interhost interval caused egg limitation in female *N. vitripennis*, we used a third GLMM, with a normal error distribution and identity link function to examine the effects of host number, interhost interval (24 or 48 h), and their two-way interaction, on the number of progeny produced by females in each oviposition (i.e., brood size). We performed all analyses in JMP Pro (SAS 2019).

## Results

The 12 F_1_ female wasps (A through L) in our experiment produced an average of 5.25 broods (3–6 broods per female), with an average of 59 F_2_ progeny per brood (17–98 offspring per brood; 63 total broods; 3,696 offspring). Each F_1_ female produced at least one mixed-sex brood among her six possible ovipositions indicating that all females had been successfully inseminated. In total, nine ovipositions (four within the 24 h interval group; five within the 48 h group) were unsuccessful due to the death or eclosion of the host fly pupa.

**Fig. 1.**
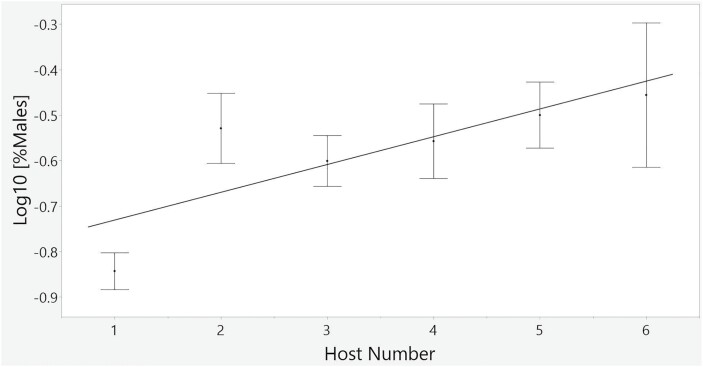
The effect of host number on log 10-transformed sex ratios in *Nasonia vitripennis.* Daily oviposition (i.e., host number) by females appears to positively affect the average brood sex ratio in *Nasonia vitripennis* (F_[1,1]_=19.42; *P* < 0.0001). Each error bar represents one standard error of the mean.

Our GLMM to examine the effect of host number and interhost interval with all possible interactions, on the proportion of males within the brood identified a significant effect of host number on the proportion of males within families (F_1,54_ = 19.42, *P* < 0.0001), but no effect of interhost interval (F_1,11_ < 0.001, *P* = 0.98). Because the interaction term (interhost interval*host number) and the main effect of inter host interval in this analysis were not significant, we removed them and performed the analysis again, using only the main effect of host number from our model. This simplified model was a better fit compared to the full model, and supported host number as a significant effect on brood sex ratio (F_[1,55]_=19.42; *P* < 0.0001, Fig. 1; Table 1). Female identity, expressed as a random effect within the model, was not significant (*P* = 0.09, Table 2). The fraction of the total variance in brood sex ratio that existed among females was less than 30% (V_sexratio(within)_ = 0.037, V_sexratio(among)_ = 0.0159; *N* = 12 females, 63 broods, 3,696 progeny), indicating that over 70% of the variation in brood sex ratio existed within females and therefore that particular females had not disproportionately contributed to our sex ratio estimates. We found no evidence that our sex ratio data were overdispersed relative to a Poisson distribution (*P* = 1.0, Table 3).

**Table 1.** Effect of interhost interval and host number on offspring sex ratios

Term	DF numerator	DF denominator	Estimate	Standard error	*F*	*P*-value
Interhost Interval	1	11	0.002321	0.128	0.0003	0.986
Host Number	1	54	0.182364	0.041	19.423	<0.0001*
Interhost Interval*Host Number	1	54	−0.02425	0.041	0.343	0.56

Results of a generalized linear mixed model examining the effects of interhost interval, host number, and their interaction on the brood sex ratio produced by females.

*Indicates significance at the 0.05 level.

**Table 2.** Variance in sex ratio due to female identity

Random effect	Variance ratio	Variance component	Standard error	95% lower	95% upper	Wald *P*-value
Female_ID	90.746	0.137	0.082	−0.023	0.297	0.094
Residual		0.002	0.0003	0.001	0.002	
Total		0.139	0.082	0.057	0.706	

Variation in offspring sex ratios produced by females during the experiment.

**Table 3.** Overdispersion test of sex ratio data

Goodness of fit statistic	Chi square	DF	*P*-value	Overdispersion
Pearson	8.440	59	1.000	0.143
Deviance	7.453	59	1.000	

Results of a goodness of fit test which examined the degree to which sex ratio data were dispersed relative to the Poisson distribution.

Our GLMM to determine the effect of total progeny produced by females during their lifetime on the proportion of males in females’ final oviposition was significant overall (X^2^ = 20.79, df = 1, *P* < 0.0001), supporting a positive relationship between total female fertility and sex ratio (B = 0.005, *P* < 0.0001, Table 4).

**Table 4.** Effect of total offspring production on final sex ratio

Term	Estimate (B)	Standard error	L-R ChiSquare	*P*-value
Intercept	−2.158	0.395	33.766	<0.0001*
Total offspring	0.005	0.001	20.793	<0.0001*

Results of a generalized linear model examining the effect of total offspring on the final sex ratio produced by females after a sequence of six ovipositions.

*Indicates significance at the 0.05 level.

Our GLMM to examine the effect of host number, interhost interval, and their two-way interaction on the number of offspring within each brood, was not significant (*P* = 0.76), indicating neither repeated oviposition nor interhost interval (i.e., the rate of oviposition) had induced egg limitation in females.

## Discussion

### The Effects of Host Number and Total Female Fertility

Consistent with our predictions, host number was significantly correlated with a higher proportion of males in brood sex ratios for both the 24 and 48 h interhost interval treatments. Moreover, females who produced more offspring overall, also produced more male offspring in their final brood. Both patterns are consistent with the hypothesis that sperm limitation may disrupt adaptive sex allocation by female parasitoids. Sperm limitation as a sex ratio constraint has been studied in numerous parasitoid species (e.g., Boivin 2013, reviewed in Boulton et al. 2015) including *N. vitripennis* (Velthuis et al. 1965; Boulton and Shuker 2015a,[Bibr CIT0009][Bibr CIT0015]). In laboratory populations, sperm limitation can arise in female wasps when ovipositing on multiple hosts (e.g., 14 per day in King 2000), or by mating with males whose sperm quality or quantity has been experimentally altered (e.g., Henter 2004, Boulton and Shuker 2015b, Martel et al. 2016, Chirault et al. 2019).

Boulton and Shuker (2015b) showed that female *N. vitripennis* mated with virgin males became sperm limited sooner than females paired with males who had mated multiple times prior. Females mated with virgin males appear to have higher fertility and live longer, which appears to increase their likelihood of experiencing sperm limitation. In our experiment, all females were mated with virgin males, which could have increased their egg production and sperm use in a manner described by these prior studies. This may explain why females in our experiment appeared to become sperm limited within comparatively few ovipositions (i.e., six). Indeed, females in our experiment, whose overall fertility was higher than in experiments mentioned above, produced more strongly male-biased sex ratios in their last brood. Although only 12 females were used in the present experiment, we found no evidence that the apparent increase in sex ratios could have been driven by a subset of the wasps.

### The Effect of Interhost Interval

Neither host number, nor interhost interval (24 and 48 h between ovipositions) influenced brood sex ratio or brood size. Parasitoids like *N. vitripennis* do not eclose from host pupae with their full complement of eggs, but instead continuously mature ova after leaving hosts (Pannebakker et al. 2013). Thus, reproductive success can be influenced by egg limitation if females encounter hosts at high rates (e.g., Shea et al. 1996, Rosenheim et al. 2000). However, in our experiment, because the number of offspring per brood did not differ between the two interval treatments (24 vs 48 h), over this time scale, egg limitation likely did not influence brood sex ratio in our experiment. This result suggests that when food is provided, 24 h between ovipositions provides enough time for a female to replenish her egg supply, or that females are not sensitive to this interval between oviposition bouts, and do not modify their sex ratios when pupae might seem to be scarce.

Our result differs from existing research showing parasitoids do respond to host availability through changes in egg production (Drost and Cardé 1992, Böckmann et al. 2011) and sex ratio (Sandlan 1979, Wylie 1979, Waage and Lane 1984). These studies achieved their effect with more extreme manipulations in host availability in than our experiment (e.g., 0–5 hosts/day and up to 12 d host deprivation in Drost and Cardé 1992). In Sandlan (1979), longer periods of host deprivation (up to nine days) appeared to increase production of daughters by ovipositing females. Relationships between host availability and sex ratio may be understood within the context of LMC, as low host density could imply a low density of other ovipositing females, and favor female-biased sex allocation due to reduced levels of LMC (Sandlan 1979, Werren 1984). In our experiment, the difference in host availability between the 24 and 48 h treatments had no effect on females’ reproductive strategy.

### Other Factors Influencing Sperm Depletion

If the costs of sperm depletion are high, females could attempt to mitigate their condition by mating multiple times at their natal host site (Ridley 1988, Chevrier and Bressac 2002, Jacob and Boivin 2005), or during encounters later in life (Hsu and Wu 2000, Mery and Joly, 2002, Steiner and Ruther 2009). However, in general, most female parasitoids copulate only once (i.e., monandrous), even if presented with opportunities to remate (Gordh and DeBach 1978, Ridley 1993). In wild populations, *N. vitripennis* females are mostly monandrous (Ridley 1993, Burton-Chellew et al. 2007) but are capable of evolving a polyandrous mating structure in a laboratory setting (van den Assem and Jackmann 1999, Burton-Chellew et al. 2007, Grillenberger et al. 2008). Female *N. vitripennis* appear to increase their fertility and longevity if mated with multiple virgin males (Boulton and Shuker 2015b) but incur costs to their fertility and produce excess male offspring in their broods if mated with multiple promiscuous males (Boivin 2013, Boulton et al. 2015, Boulton and Shuker 2015b).

Ejaculates from multiple males can reduce the effectiveness of sperm use by females, which may increase the proportion of males in brood sex ratios (Boulton and Shuker 2015b; Boulton et al. 2018, 2019). Opportunities for postdispersal mating are also less likely in *N. vitripennis* due to the shorter lifespan and limited dispersal ability of flightless males (King 1993, Molbo and Parker 1996). *N. vitripennis* males also develop to maturity with their full supply of sperm and do not produce more as they age (Hogge and King 1975). These males may also mate repeatedly if multiple females are present and remain eager to copulate even if their sperm supplies have been depleted (van den Assem 1986, Damiens and Boivin 2006). Thus, if females encounter males at other oviposition sites after leaving their natal host, it is reasonable to assume these males will transfer fewer sperm due to their previous mating history (Henter 2004, Damiens and Boivin 2006, Bressac et al. 2008). These characteristics in *N. vitripennis* suggest that if sperm limitation arises within a female’s lifetime, it is likely to be permanent. 

## Conclusions

Given the emphasis placed on female influences on brood sex ratio in *N. vitripennis*, we did not expect females to be highly sensitive to sperm depletion. Female *N. vitripennis* are widely thought to vary their offspring sex ratios as needed to minimize local mate competition (Werren 1980). Females do so either as primary females, producing mostly daughters when mating competition for sons is low, or as secondary females, increasing their proportion of sons in response to a surplus of females left on hosts by earlier ovipositing females. Werren (1984) reported that in the primary female’s sex ratio pattern, ‘a strongly daughter biased sex ratio is produced and sex ratio is independent of brood size’ (p. 123). Our results confirm that a female’s brood size does not affect her primary sex ratio. However, contrary to LMC predictions, females are not always able to produce strongly female biased broods in primary ovipositions. Instead, our results suggest that the primary female pattern may be limited by an adequate supply of sperm, which, at least in our study, became depleted in females within six ovipositions. Overall, our results suggest that, when mating structure and initial sperm resources are considered, sex ratio adjustment by parasitoids like *N. vitripennis* could become less flexible later in the species’ lifecycle.

Consistent with prior research, our results support the hypothesis that oviposition-mediated sperm limitation, but not egg limitation, reduces females’ ability to bias their offspring sex ratios toward daughters. Moreover, this limitation becomes important within relatively few ovipositions, regardless of individual fecundity. We suggest that this simple relationship may lead to a better understanding of maternal influences on brood sex ratios in nature.

## Dryad Data Location

https://doi.org/10.5061/dryad.mw6m905zw.
